# The microRNA212 regulated PEA15 promotes ovarian cancer progression by inhibiting of apoptosis

**DOI:** 10.7150/jca.32886

**Published:** 2020-01-13

**Authors:** Yonghong Luo, Chuanchuan Fang, Lan Jin, Huafeng Ding, Yuanyuan Lyu, Guantai Ni

**Affiliations:** 1 Department of Obstetrics and Gynecology, The First Affiliated Hospital of Wannan Medical College, Wuhu, AnHui, 241001, P.R. China; 2 Wannan Medical College, Wuhu, AnHui, 241001, P.R. China

**Keywords:** PEA15, miR212, Proliferation, Apoptosis, OC

## Abstract

PEA15 (Proliferation And Apoptosis Adaptor) is a 15kDa multifunctional phosphoprotein involved in various essential biological processes such as proliferation and apoptosis of cancer cells. Previous studies have demonstrated that PEA15 can promote the progression of many malignancies. In the present study, the expression of PEA15 in ovarian cancer and normal tissues analyzed in several databases and PEA15 was found to be significantly up-regulated in OC tissues compared to normal tissues. Immunochemical assays performed using 171 OC tissue specimens proved that the expression of PEA15 was remarkably positively correlated with the FIGO stage and associated with histologic subgroups of ovarian cancer. IHC assay for the two phosphorylation sites of PEA15 S116 and S104 was also performed. PEA15 high expression predicted a poor prognosis in OC patients analysed from K-M plot dataset. In addition, we proved knockdown of PEA15 inhibits OC cell proliferation and induces cell apoptosis by Bcl2 downregulation and Bax and cleaved Caspase-3 upregulation. Overexpression of PEA15 promotes the proliferative capacity of OC cells. Moreover, this study first discovered PEA15 expression in OC can be negatively regulated by microRNA212. Overexpression of miR-212 in ovarian cancer cells could cause downregulated the expression of PEA15 expression. Overexpression of miR-212 was found to exerted similar effects on the proliferation, and apoptosis of the ovarian cancer cells as that of PEA15 suppression. Additionally, overexpression of PEA15could at least partially abolished the effects of miR-212 on the proliferation, and apoptosis of ovarian cancer cells. In conclusion, our findings revealed PEA15 appears as a novel predictive biomarker, thus providing a valuable therapeutic target in OC treatment strategy.

## Introduction

Ovarian cancer (OC) has the highest mortality rates among gynecological malignancies and is the 5^th^ cause of cancer-related deaths among women 1. Current therapeutic methods, such as surgery and chemotherapy are not satisfactory, especially for advanced stage of the disease (FIGO stages III/IV) 2, it often accompanied by malignant proliferation, extensive invasion, and lymphatic metastasis at the time of diagnosis 3. The carcinogenesis of OC is complex as it is influenced by genetic alterations of oncogenes and tumor suppressor genes 4. The molecular mechanisms regulating the varying degree of proliferation and cell death resistance of OC are largely unknown. Therefore, it is important to explore novel molecular markers that regulate the proliferation and apoptosis of OC cells.

PEA15 (Proliferation and Apoptosis Adaptor Protein 15) consists of 130 amino acid residues. It performs many functions involved in broad-spectrum resistance to apoptosis 5. Prior research reveals that it blocks Ras-mediated inhibition of integrin activation and modulates the ERK MAP kinase cascade, it also decreases RPS6KA3 activity by retaining it in the cytoplasm and suppresses TNFRSF6- and TNFRSF1A regulated CASP8 activity and apoptosis 6. In recent years, studies have found that the expression of PEA15 is closely related to several types of cancers 7. For instance, PEA15 protein was found to be highly in esophageal carcinoma and over expression of PEA-15 shortened the average survival time of patients 8. Existing literature indicates that PEA15 is involved in the tumourigenesis of breast cancer 9, thyroid cancer 10, colorectal cancer 11 , amongst others.

In our study, we analyzed the expression of PEA15 in OC tissues and the corresponding normal tissues. The clinicopathological significance of PEA15 was determined by examining its expression in OC tissues by immunohistochemical staining. In addition, its biological impact on OC cell lines and its upstream regulatory miRNA were explored.

## Material and Methods

### In silico analysis using the The Cancer Genome Atlas (TCGA), Gene Expression Omnibus (GEO), Oncomine and KM plotter databases

To investigate the expression pattern of PEA15 in OC, we used the 3 datasets TCGA (https://tcga-data.nci.nih.gov/, www.oncomine.com/) and GEO (https://www.ncbi.nlm.nih.gov/gds/). We further analyzed the OS and PFS of over 1000 patients from K-M plot dataset (http://kmplot.com/analysis/). Data was collected and analysed using R 3.0.2 software. PEA15 gene expression in OC tissues and normal tissues was compared using a previously described calculation method.

### Clinical tissue samples

Tissue microarrays from 71 OC samples were obtained from the First Affiliated Hospital of Wannan Medical College (Wuhu, China) between December, 2011 and June, 2015. Additional 20 freshly-frozen OC tissues and matched non-cancerous tissues were obtained from patients who underwent surgical resection at the First Affiliated Hospital of Wannan Medical College. None of these patients had received radiotherapy, chemotherapy, hormone therapy or other related anti-tumor therapies prior surgery. None of them had was diagnosed with other cancers. Postoperative adjuvant therapies were administered, according to standard schedules and doses. All participants who provided samples gave written informed consent, and the experiments were approved by the local ethics committee of the First Affiliated Hospital of Wannan Medical College.

### Tissue microarrays construction

Paraffinized tissue blocks of samples from 171 cases were stained with hematoxylin-eosin to confirm the diagnoses and were marked at fixed points with typical histological characteristics under a microscope. Two 1.5mm cores per donor block were transferred into a recipient block tissue microarray, and each dot array contained less than 160 dots. Three-micron-thick sections were cut from the recipient block and transferred to glass slides with an adhesive tape transfer system for ultraviolet cross-linkage.

### Immunohistochemistry (IHC) staining

IHC staining was performed according to a previously described procedure 21. Rabbit polyclonal PEA15 antibody (1:200, Proteintech, USA), PEA-15 with phosphorylated Ser104 (PEA-15-pSer104), or PEA-15with phosphorylated Ser116 (PEA-15-pSer116)(Santa Cruz Biotechnology, Inc., Shanghai, China) were used. All sections were observed and imaged using a microscope (Axio Imager: Carl Zeiss). Scoring was carried out according to the ratio of positively stained cells (0 = negative, 1 = 1-25% of cells, 2 = 26-50% of cells, 3 = 51-75% of cells and 4 =76-100% of cells were stained) and staining intensity (no staining scored 0, weakly staining scored 1, moderately staining scored 2 and strongly staining scored 3). The final score of PEA15 was presented as the proportion of positive cell score × staining intensity score as follows: “-” for a score of 0-2, “+” for a score of 3-5, “+ +” for a score of 6-9 and “+ + +” for a score of > 9. Low expression was defined as a total score < 6 and high expression as a total score ≥ 6. These scores were determined in a blinded manner by two senior pathologists and presented as the mean percentage of the two scores.

### Cell culture

The immortalized human OC cell lines were obtained from the Cell Bank of the Chinese Academy of Sciences (Shanghai, China). Cells were routinely cultured with RPMI 1640 medium (Gibco, Beijing, China) supplemented with 10% fetal bovine serum and 1% antibiotics (100 μg/mL streptomycin and 100 units/mL penicillin) at 37˚C in an incubator with a 5% CO2 atmosphere.

### RT-PCR

Total cellular mRNA was isolated using TRIzol reagent (Takara) and then reverse transcribedwith the PrimeScript RT-PCR kit (Takara) according to the protocol provided. The mRNA expression of PEA15 (sense: 5'- GGAGAGCCACAACAAGCTG-3'; anti-sense: 5'- CCATAGTGAGTAGGTCAGGACG-3') was determined by real-time PCR using SYBR Premix Ex Taq (Takara) and a 7500 real-time PCR system (Applied Biosystems) at the following cycling settings: one initial cycle at 95˚C for 10 s, 40 cycles at 95˚C for 5 s, followed by 30 s at 60˚C. Data were normalized to 18s expression (sense: 5'-TGCGAGTACTCAACACCAACA-3'; anti-sense: 5'-GCATATCTTCGGCCCACA-3') and are presented as the average of 3 repeated experiments, and were calculated with the 2-ΔΔCT method.

### Western blotting

Total cellular proteins were extracted using a total protein extraction kit (Beyotime, China). Cell lysates were separated by 10% SDS-PAGE gel electrophoresis and transferred to a nitrocellulose membrane. The membranes were blocked with 5% nonfat milk and incubated with the primary antibodies and then incubated with species-specific secondary antibodies. Image J software was used to analyze the results. The following antibodies were used at the indicated concentrations PEA15 (1:1000, Proteintech, USA), Bcl2 (1:1000, CST, USA), Bax (1:1000, CST, USA), Cleaved caspase-3 (1:1000, CST, USA), ERK(1:1000, Abcam, USA), p-ERK(1:1000, Abcam, USA), IRDye680 anti-mouse (1:20000, LI-COR) and IRDye800 anti-rabbit (1:10000, LI-COR). GAPDH (1:1000, CST, USA) was used as a control to confirm equal loading of protein samples.

### Transfection

Lipofectamine 3000 (Invitrogen), stable transfections were constructed and used according to the manufacturer's instructions. The shPEA15, Lenti-PEA15, shmiRNAs and miRNA mimics plasmid were synthesized by Yazai biological company (Shanghai, China). Cells were infected with shmiR-212 lentiviral/miR-212-mimics lentiviral or shPEA15/Lenti-PEA15 lentiviral, and then harvest-ed at 48 h post infection, the gene silencing effects were verified by RT-PCR.

### Luciferase assays

The 3'-untranslated regions (UTRs) of PEA-15 genes were synthesized, and then inserted into the psiCHECK-2 report plasmid using the HindIII and SpeI sites located downstream from the stop codon of luciferase. To perform luciferase assays, OVCAR8 cells were co-transfected with plasmids and miR-212 using Lipofectamine 3000 according to the manufacturer's protocol. At 48h after transfection, firefly and Renilla luciferase activities were measured by the Dual-Luciferase Reporter Assay System (Promega, Massachusetts, U.S.). The experiments were performed three times.

### Cell viability assay (CCK8 assay)

NC and shPEA15 OC cells were seeded on 96-well plates at a density of 2000-3000 cells per wellwith 100μL medium containing 10% serum. Five wells were prepared for each cell. Cell Counting Kit-8 solution (CCK-8, Dojindo, Japan) solution (10μL) was added to each well after 0, 24, 48, 72 and 96h. Cell viability was measured using a microplate reader (BIO-TEK) at the absorbance of 450 nm. This experiment was performed twice for each time point.

### Plate Colony Formation Assay

Plate colony formation (PCF) assay was carried out using six-well plates. NC, shPEA15, Lenti-PEA15 OC cells (1 × 10^3^) were seeded in each well with 2 ml DMEM supplemented with 10% FBS. The culture medium was changed every 3 days. After 14 days, the resulting colonies were fixed with methanol at -20°C for 5 min, and then stained with crystal violet. Only clearly visible colonies (diameter > 50 μm) were counted.

### *In vivo* tumor xenograft model

Six-week-old male nude (nu/nu) mice (SLAC, Shanghai, China) were injected subcutaneously in the right flank with stable clones of OVCAR8 cells at a density of 5×10^6^, infected with OVCAR8-NC or OVCAR8-sh3 in 100μL sterilized phosphate-buffered saline. Each group contained 5 mice, and the tumor weights were calculated and recorded. Six weeks later, all mice were sacrificed and their tumors were dissected, fixed with phosphate-buffered neutral formalin, embedded in paraffin and prepared for standard histological examination. All mice experiments upon and handling were performed in accordance to protocols approved by the East China Normal University Animal Care Commission.

### Cell apoptosis analysis

In the apoptosis assay, cells were stained with propidium iodide and Annexin V-fluorescein isothiocyanate (BD Pharmingen) in accordance with the manufacturer's instructions. Briefly, cells were washed with PBS and resuspended in 1× Binding Buffer, then, 5 μl FITC Annexin V and 5 μl PI were added to 100 μl of the cell suspension and incubated for 15 min in the dark. After incubation, 400 μl 1× Binding Buffer was added. Apoptosis was analyzed by FACS using the Cell-Quest software. Annexin V-FITC-positive and PI-negative cells were apoptotic.

### Statistical analysis

Data analyses were performed using SPSS version 21.0 (IBM Corporation) and GraphPad Prism7 (San Diego, CA) software. Clinicopathological characteristics were analyzed by the chi-square test. Differences between groups were compared using a two-tailed Student's *t* test. All *P*-values were determined from 2-tailed tests and differences with a *P*-value < 0.05 were considered to be statistically significant.

## Results

### The expression level of PEA15 is significantly elevated in ovarian cancer tissues and is correlated with clinicopathological features

**Figure 1:**
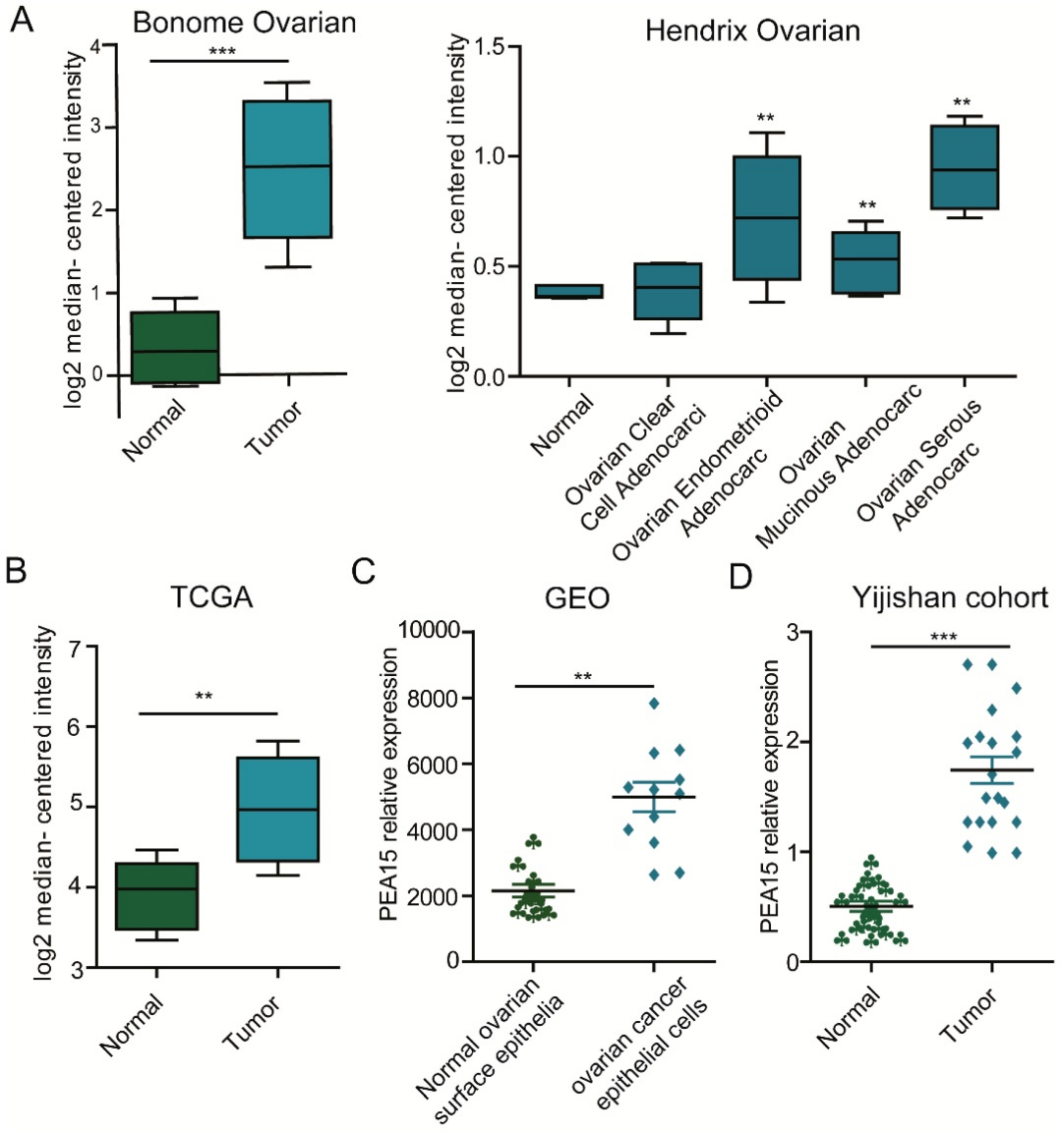
** The expression of PEA15 is significantly upregulated in ovarian cancer tissue. A:** The mRNA expression of PEA15 is upregulated in different tumor types compared with the normal tissues based on the Oncomine dataset. **B-C:** The mRNA expression of PEA15 is upregulated in tumor compared with non-tumor tissue as revealed by the TCGA and GEO datasets. **D:** PEA15 mRNA expression is elevated in 20 paired OC tissues and normal tissues obtained from the first affiliated hospital of Wannan Medical College.

**Figure 2:**
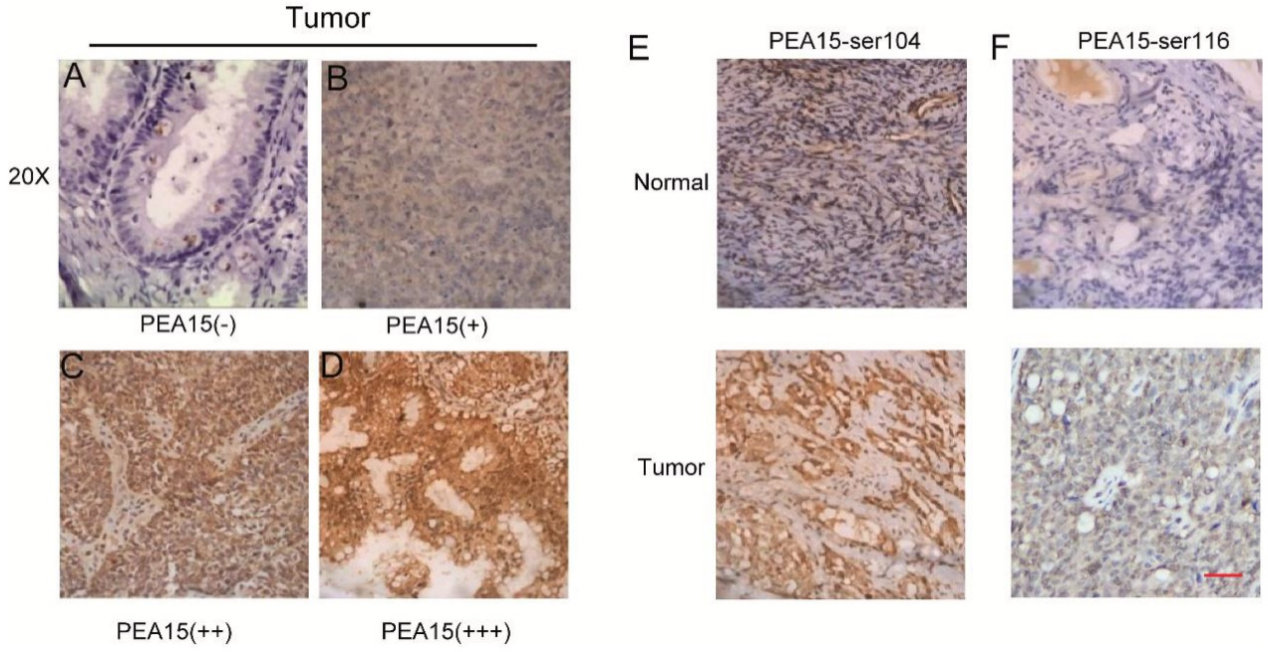
** PEA15 expression in OC tissue samples and immunohistochemical staining of two phosphorylation sites S116 and S104 of PEA15in normal and tumor tissues.** A-D: Representative images of PEA15 expression in OC are shown at 200x magnification. A: OC, scored as (-); B: OC, scored as (+); C: OC, scored as (++); D: OC, scored as (+++). E: Normal ovarian tissue with negative PEA15-Ser104 staining and tumor tissue with positive PEA15-Ser104 staining. F: Normal ovarian tissue with negative PEA15-Ser116 staining and tumor tissue with positive PEA15-Ser116 staining.

To determine the expression status of PEA15 in human OC tissues, data was retrieved from in three independent microarray datasets, including the TCGA (The Cancer Genome Atlas), GEO (Gene Expression Omnibus) and Oncomine databases (**Figure 1A-C**), exported and used to analyze the expression level of PEA15. The results revealed that the expression level of PEA15 was significantly increased in tumor tissues compared with normal tissues in all independent datasets. We further detected the mRNA expression level of PEA15 in 20 pairs of OC and adjacent normal tissues. Consistent with the data from the TCGA, GEO and Oncomine databases, the expression level of PEA15 was remarkably up-regulated in tumor tissues compared to adjacent normal tissue. We then analyzed the expression of PEA15 protein in 20 normal ovarian tissues and 171 cases of malignant carcinoma tissues by immunohistochemistry (**Figure 2**). Analysis of the relevance of PEA15 expression with clinicopathological features revealed that PEA15 protein expression was differentially expressed (*P* = 0.012) among the histologic subgroups of ovarian cancer (endometrioid, serous, clear cell and mucinous). High expression of PEA15 was strongly related with adverse clinicopathological parameters of EOC, including FIGO stage and lymph node metastasis. Moreover, patients with high stage (III-IV vs I-II) (*P* = 0.001) and lymph node metastasis (*P* = 0.005) had higher expression of PEA15 (As shown in **Table 1**).

### The expression of PEA15 is a poor prognostic factor for patients with OC

**Figure 3:**
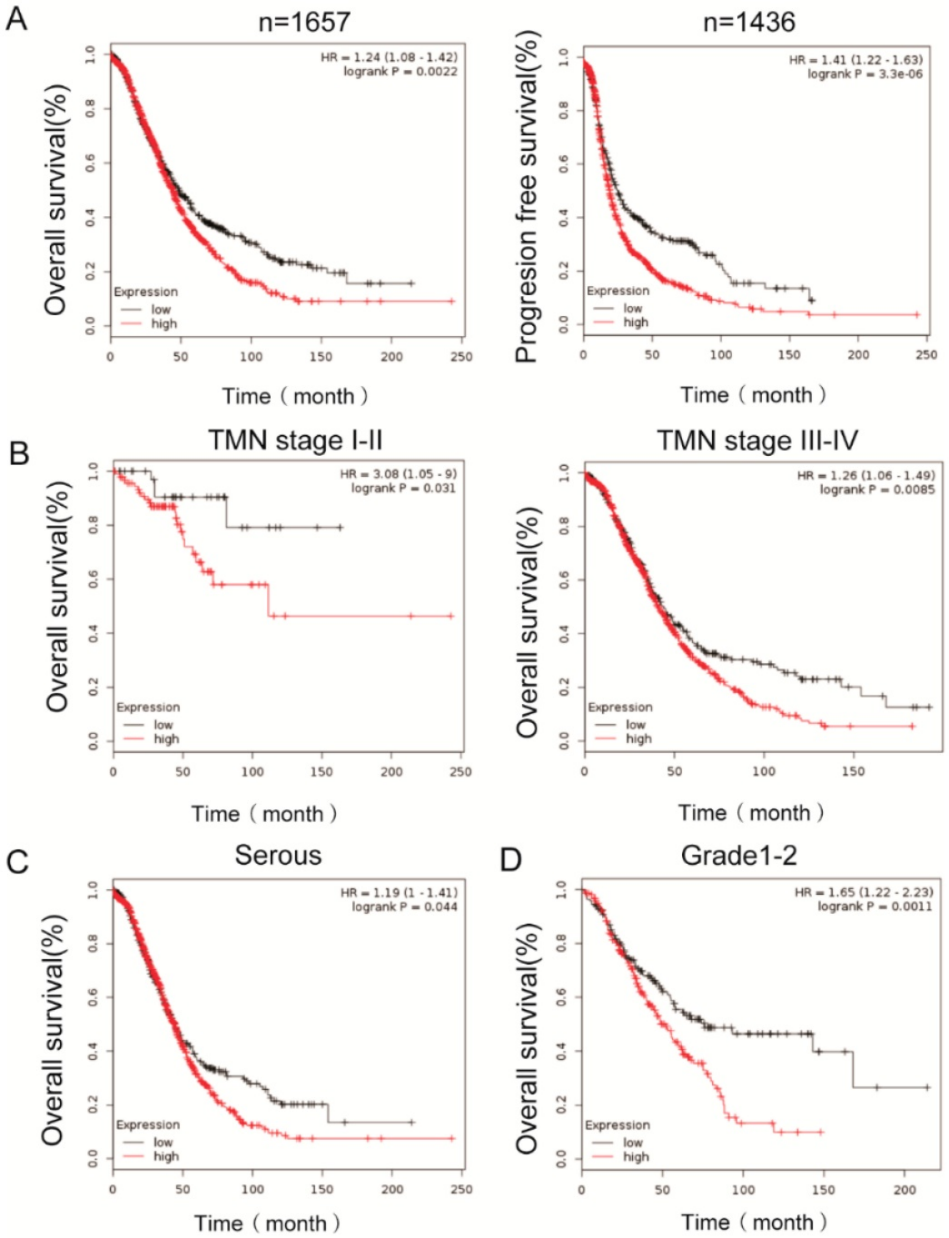
** Kaplan-Meier analysis of overall survival in OC patients based on K-M plotter dataset. A:** PEA15 expression is negatively correlated with overall survival (OS) and progression free survival (PFS) in OC patients; **B-D:** Correlation between PEA15 expression and overall survival is independent of clinical stage (**B**), type (**C**) and Grade (**D**).* P*-values were calculated by log-rank test.

To determine the clinical significance of PEA15, its prognostic value in OC was examined by analyzing survival data of more than 1000 ovarian cancer patients from K-M plotter dataset. The results demonstrated that high expression of PEA15 was inversely associated with overall survival (OS) (n=1657, *P* =0.0022, **Figure 3A**) and progression free survival (PFS) (n=1436, *P* < 0.001, **Figure 3B**), indicating that patients with higher PEA15 levels will have significantly poorer prognosis than those with lower PEA15 levels. Moreover, high expression of PEA15was remarkably associated with poor OS of patients, regardless of the TNM stage (**Figure 3C-D**), type (**Figure 3E**) and Grade (**Figure 3F**).

### Silencing of PEA15 suppresses OC cell proliferation *in vitro* and tumor growth *in vivo*

**Figure 4:**
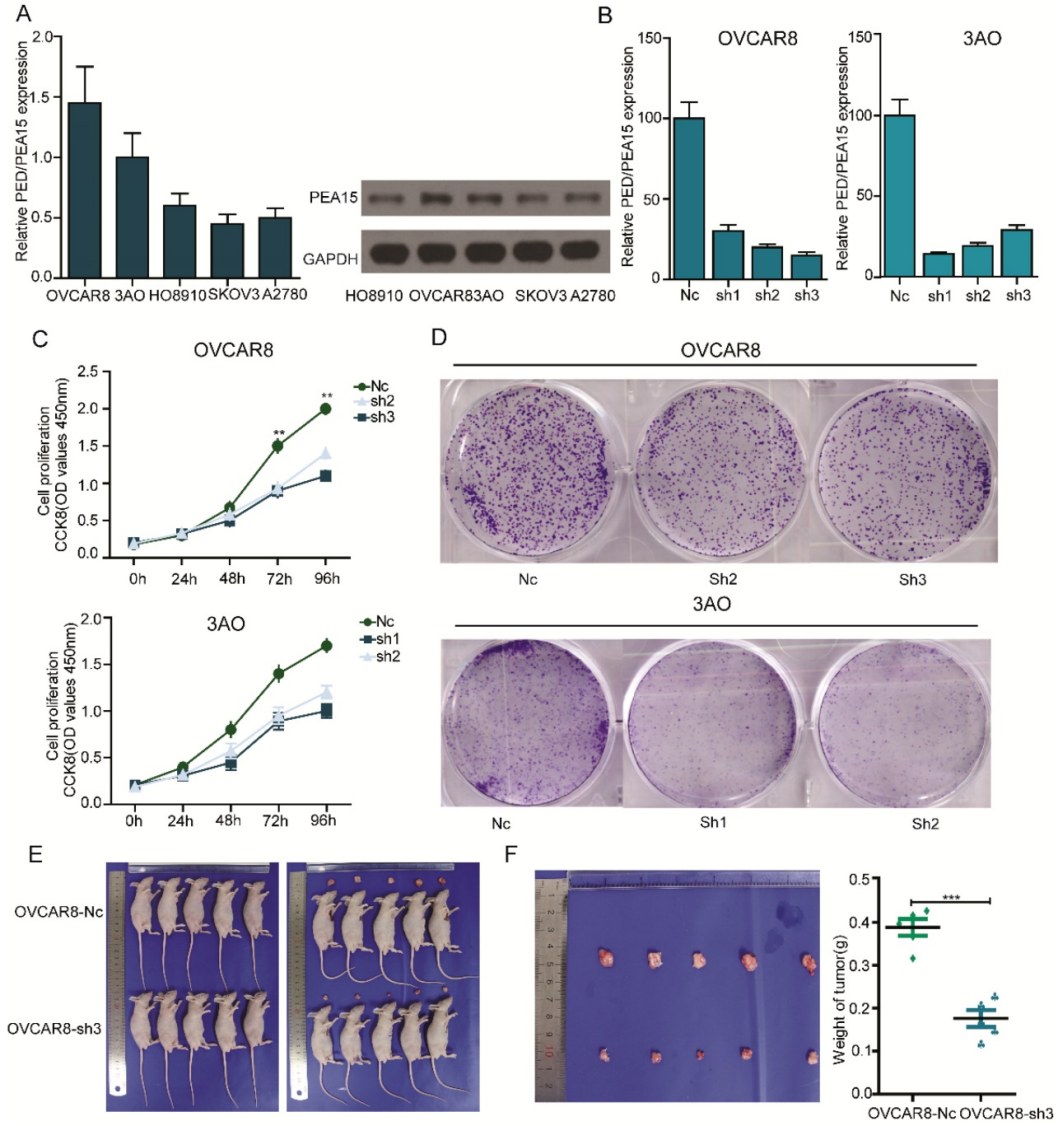
** Silencing of PEA15 suppresses ovarian cancer cell proliferation *in vitro* and tumor growth *in vivo*. A:** RT-PCR and western blotting experiments showing the PEA15 expression at mRNA and protein level in 5 OC cell lines. **B:** PEA15 knockdown efficiency was confirmed by RT-PCR in OVCAR8 and 3AO cells. **C:** The cell proliferation of Nc and sh-groups in OVCAR8 and 3AO cells were evaluated by CCK8 assay at 0, 24, 48, 72, 96h. The results showed that knockdown of PEA15 significantly inhibited the proliferation of OVCAR8 and 3AO cells *in vitro* (P<0.01). **D:** Plate colony formation assay of OVCAR8/PEA15-sh and 3AO/PEA15-sh and Nc cells on regular culture plates after 14 days of culture. Relative colony numbers of PEA15-sh cells were significantly lower than that of Nc cells. **E:** Photographs of tumors from mice inoculated with OVCAR8/Nc and OVCAR8/sh3 cells. **F:** Tumor weights of Nc and sh3 groups shown in figure 4F, n = 5

To gain a better understanding of the biological function of PEA15 in OC, we detected the expression of PEA15 in 5 OC cell lines (OVCAR8, 3AO, HO8910, SKOV3, A2780) using RT-PCR and western blotting (**Figure 4A**). Two OC cell lines with relatively high PEA15 expression (OVCAR8, 3AO) were used in the loss-of-function experiment. The two cell lines were transfected with shPEA15-(1, 2, 3), designated as sh1, sh2 and sh3, or a mock vector, which was labeled as Nc. The silencing effects were validated by RT-PCR (**Figure 4B**). The results showed that PEA15 expression was significantly decreased after sh2 and sh3 in OVCAR8 cells and 3AO by sh1 and sh2. To further explore the effect of PEA15 on OC cell growth, cell proliferation of the transfected cell lines was examined by both CCK8 and plate colony formation assays. The results of both assays showed that reducing the expression of PEA15 significantly suppressed the proliferation ability of OVCAR8 and 3AO cells *in vitro* (*P*<0.001) (**Figure 4C-D**). Moreover, the inhibitory effect of silencing PEA15 on proliferation was verified *in vivo* again by subcutaneously inoculating OVCAR8-sh3 and Nc cells into nude mice. Six weeks later, tumors derived from the sh3 cells were significantly smaller than those derived from Nc cells (**Figure 4E**). The average tumor weight in 5 OVCAR8-Nc mice was 0.41 g ± 0.06 g, in contrast to 0.16 ± 0.03 g in OVCAR8-sh3 nude mice (**Figure 4F**).

Taken together, these findings demonstrated that silencing of PEA15 in the ovarian cancer cells suppressed both cell proliferation *in vitro* and reduced tumorigenesis *in vivo*.

### Overexpression of PEA15 promotes OC cell proliferation *in vitro*

To further explore the biological role of PEA15 in OC, two OC cell lines with relatively low PEA15 expression (A2780 and SKOV3) were used to perform gain-of-function tests. The two cell lines were transfected with Lenti-PEA15 or a mock vector labeled as Ctrl. The overexpression efficiency was confirmed by RT-PCR (**Supplementary Figure 2A**). The results showed that PEA15 expression levels were significantly increased by Lenti-PEA15 in A2780 and SKOV3 cells. CCK8 assay and plate colony formation assay also performed to detect cell proliferation rate of Lenti-PEA15 cells and Ctrl cells. Both experiments demonstrated that overexpression of PEA15 significantly enhanced the proliferation capacity of A2780 and SKOV3 cells *in vitro* (*P*<0.01) (**Supplementary Figure 2B-C**).

### Silencing of PEA15 decreases the expression of pERK in ovarian cancer cells

In this study, we further detected the expression of ERK and pERK after the expression of PEA15 was knocked down in ovarian cancer cells by western-blot analysis. The result demonstrated that PEA-15 silencing did not change the expression of ERK, but it decreased the expression of pERK in OVCAR8 and 3AO cells (**Supplementary Figure 3**).

### Silencing of PEA15 induces apoptosis of OC cells by regulating the expression of apoptosis related proteins

**Figure 5:**
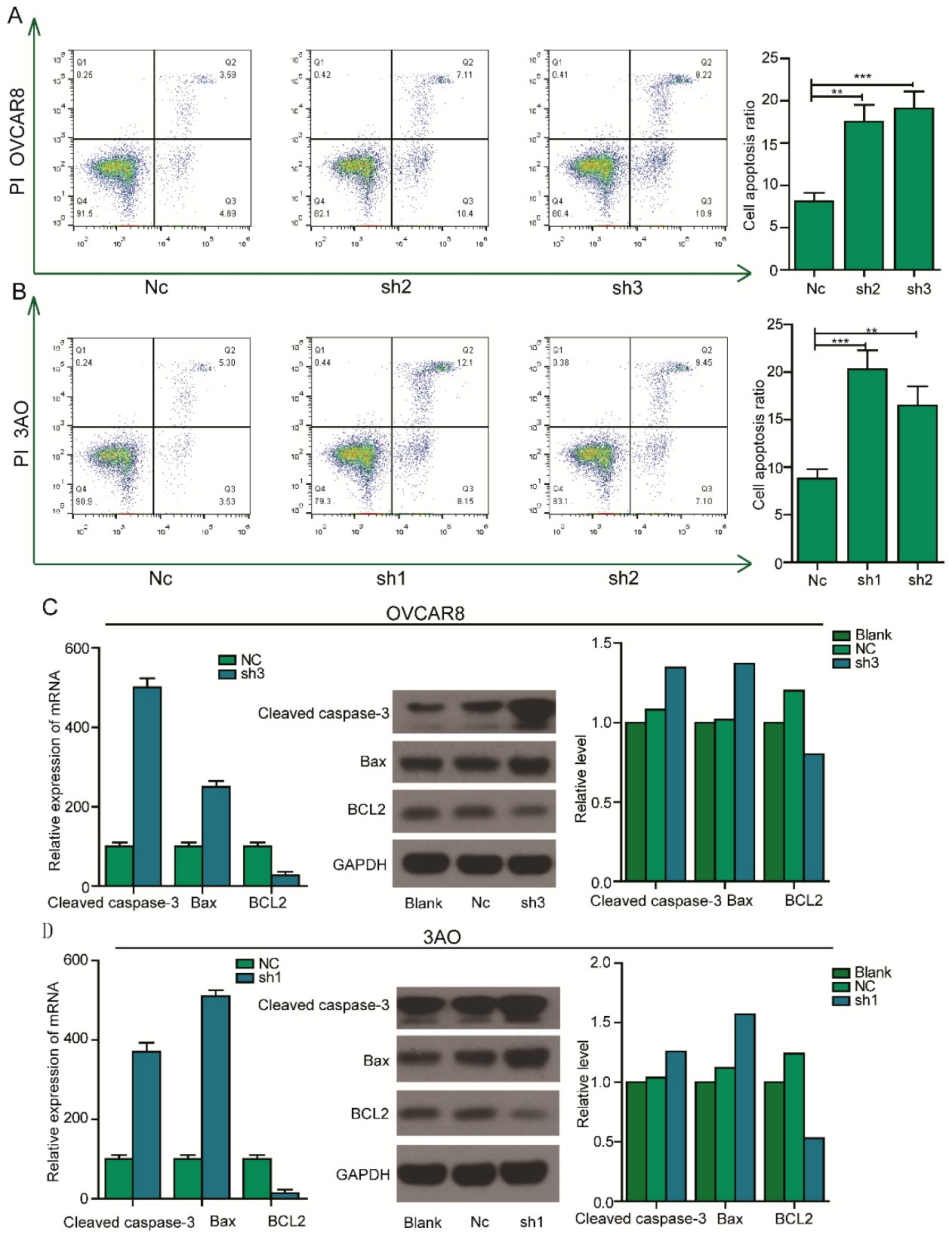
** Effect of PEA15 on cell apoptosis in OC cells. A-B:** Downregulation of PEA15 significantly increased apoptosis in OVCAR8 and 3AO cells, the statistical results are shown on the left (*P < 0.05, **P < 0.01). **C-D:** The expression of Bcl2, Bax and cleaved caspase3 were determined by RT-PCR and western blotting analysis in OVCAR8 and 3AO cells.

The inhibition of cell proliferation by silencing of PEA15 prompted us to further assess the effects of PEA15 on apoptosis. To test this possibility, PEA15 silenced OC cells were examined by flow cytometry after Annexin V and PI double staining. The result showed that the rate of apoptosis of PEA15-silenced OVCAR8 cells (PEA15-Sh2, PEA15-Sh3) was significantly higher compared with that of OVCAR8 cells transfected with Nc (8.2% ± 2.1% vs. 17.5% ± 3.6%, 19.1% ± 2.2%, respectively;* P* <0.05,** Figure 5A**). Consistent with this finding, results showed that downregulation of PEA15 in 3OA cells increased apoptosis rate compared with Nc cells (8.8% ± 1.5% vs. 20.2% ± 1.1%,16,5% ± 3.4%, respectively;* P* < 0.05; **Figure 5B**). To explore the mechanism by which PEA15 influenced the apoptosis process, the expression of several apoptosis-related proteins were detected by western blotting. It was found that low PEA15 expression in OVCAR8 and 3OA cells decreased the expression level of anti- apoptosis protein Bcl2, but increased the expression of pro-apoptosis proteins Bax and cleaved caspase-3 (**Figure 5C**).

### PEA15 is negatively regulated by miR212 and overexpression of PEA15 abolishes the effects of miR212 in OC cell lines

**Figure 6:**
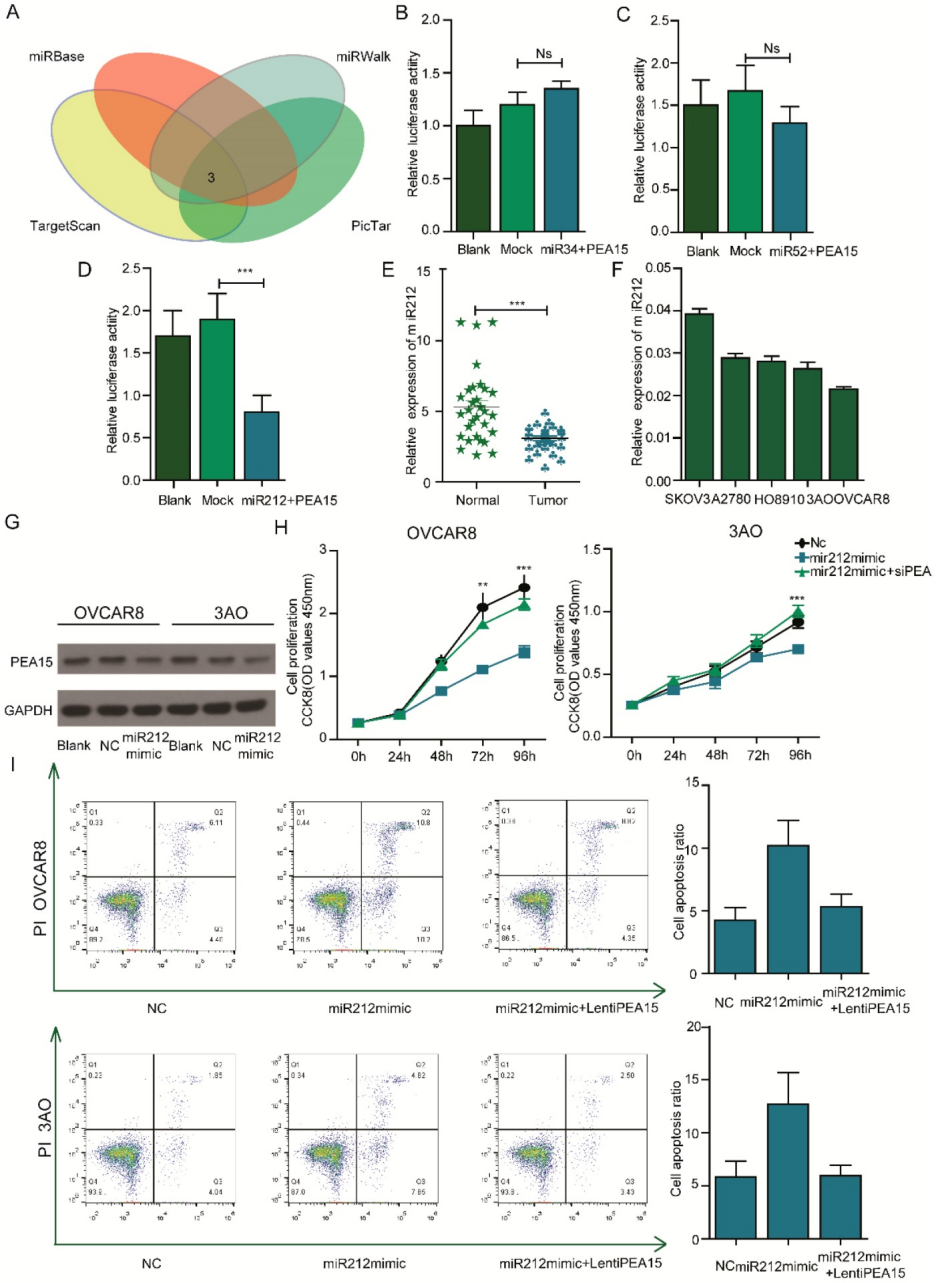
** PEA15 is negatively regulated by miR212 and silencing of PEA15 abolishes the effects of miR212 in OC cell lines. A: A**nalysis of TargetScan, miRwalk, picTar and miRBse datasets identified 3 miRNAs as potential target of PEA15. **B-D:** Effect of 3 miRNAs on the luciferase activity of WT PEA-15 3′UTR in OVCAR8 cells by luciferase reporter assay. Blank PEA-15 negative vector, Mock 3 miRNAs plus PEA-15 negative vector. Error bars mean ± SEM. **E:** The expression of miR212 is downregulated in ovarian tumor tissues compared with normal tissues. **F:** The expression of miR212 in 5 ovarian cancer cells. **G:** The protein expression of PEA-15 is down-regulated when miR-212 is overexpressed. **H:** Effect of reintroduction of PEA-15 on miR-212-induced cell proliferation by CCK-8 assay (*P* < 0.01 with two-way ANOVA). **I:** Effect of reintroduction of PEA-15 on miR-212-induced cell apoptosis by Flow analysis (*P* < 0.01 for both groups).

To determine the microRNAs that regulate PEA15, the online softwares (Target Scan, miRbase, miRwalk, PicTar) were used. Several miRNAs such as miR34, miR52 and miR212 were identified to be upstream regulators of PEA15 (**Figure 6A**). To identify the specific miRNA that regulate PEA15, a luciferase reporter assay was performed. The results showed that miR212 negatively regulated the expression of PEA15 (**Figure 6B-D**). The expression of miR212 in ovarian cancer and normal tissues was investigated by RT-PCR (**Figure 6E**). The results indicated that miR212 was significantly downregulated in cancer tissues relative to normal tissues. In OC cell lines and two OC cell lines with relatively high PEA15 expression (OVCAR8, 3AO), the expression of miR212 was relatively low (**Figure 6F**). To examine the interaction between miR212 and PEA15, further tests were performed. First, we demonstrated that overexpression of miR212 sharply decreased the expression of PEA15 in both OVCAR8 and 3AO cell lines by western-blot (**Figure 6G**). Next, we investigated the effects of miR212 on the proliferation and apoptosis of OC cell lines. The results showed that overexpression of miR212 significantly reduced cell proliferation activity and remarkably increased apoptosis of OVCAR8, 3AO cell lines. Restoration of PEA15 abolished the effects of miR212 on OC cell lines (**Figure 6H-I**).

## Discussion

Ovarian cancer is one of the three major gynecological malignancies in women 12, 13. The mortality due to ovarian cancer is higher than that of endometrial cancer and cervical cancer even though its incidence rate is relatively lower 14. So far, multiple molecular changes have been found to contribute to the tumorigenesis in OC 15-17.

PEA15, which is a small phosphoprotein previously found to be a negative regulator of apoptosis, has been identified as an oncogene or tumor suppressor gene in the occurrence, development, and growth of tumors 18, 19. So far, a number of studies have revealed that high expression of PEA15 protein influences the characteristics of tumors, differentiation degree, prognosis of cancer, and response to chemoradiotherapy 20. Several studies have shown that PEA15 has a pro-survival and tumorigenic PEA15 in skin cancer 21. Similarly, another study revealed the gain of PEA15 expression antagonized and its loss enhanced CCT68127-mediated growth inhibition in lung cancer 22. A previous study reported that PEA15 was highly expressed in the cytoplasm and nucleus of tumor cells and induced autophagy in human cells 7. For instance, it was reported that PEA-15 induces autophagy in human ovarian cancer cells and is associated with prolonged overall survival. In contrast, our research provides a new perspective on the role of PEA15 in ovarian cancer based on large-scale surveys from authoritative databases and subsequent biological experiments. The following results were found in this study:

The two major objectives of cancer research is to identify key factors and biological mechanisms that lead to poor outcomes. Based on the analysis of ovarian cancer microarray data from Oncomine, GEO and TCGA databases, we found that mRNA expression of PEA15 was significantly up-regulated in ovarian cancer cells compared to normal tissues. The expression of PEA15 in 171 OC samples was detected. We found that the expression of PEA15 in OC tissues was positively related to the clinical stage and histologic grade of ovarian cancer, indicating that PEA15 might participate in the development of epithelial ovarian cancer. Survival analysis of over 1000 OC patients from Kaplan-Meier plotter database and the result indicated that patients with high PEA15 expression exhibited a remarkably shorter survival duration than those with low PEA15 expression levels, suggesting that PEA15 may participate in the development of OC.

Other studies have reported that the activity of PEA-15 is highly dependent on the phosphorylation status of Ser104 and Ser116 23. Unphosphorylated PEA-15 can act as a tumor-suppressor, whereas phosphorylation fine-tunes its interaction with other factors to promote tumor development 24. In our study, the phosphorylation status of PEA15 in OC was detected by IHC. Results revealed that PEA15-pSer104 and PEA15-pSer116 were highly expressed in malignant carcinoma. In summary, our data indicate that high expression levels of unphosphorylated PEA15, PEA15-pSer104, and PEA15-pSer116 may contribute to histological type and FIGO stage. Further studies should explore the correlation between the phosphorylation status of PEA15 at Ser104 and Ser116 sites and clinic pathological features of Ser104 and Ser116 in ovarian carcinoma.

To further elucidate the biological effects of PEA15 on OC cell lines, we established stable PEA15 silencing using 3AO cell clone and OVCAR8 cell clone to perform loss of function experiments. Our results showed that the proliferative ability of OC cells was significantly decreased *in vitro* as measured by CCK-8 assay. A nude mouse xenograft model was built to verify the effects of PEA15 silencing *in vivo*. Similarly, PEA15 silencing decreased proliferation activity of OC cells *in vivo*. In conclusion, our data demonstrated that PEA15 promoted the proliferation of OC cells.

Apoptosis, or programmed cell death, is involved in various biological processes during tumor pathogenesis 25, 26. The imbalance between cell apoptosis and proliferation leads to cancer progression 27-29. In our experiments, we found that silencing PEA15 triggered apoptosis in OVCAR8 and 3AO cell lines, indicating that PEA15 inhibits apoptosis in human OC. Moreover, we found that the effects of silencing of PEA15 on apoptosis were associated with alteration in the expression of the Bcl2 family proteins. The Bcl2 family proteins are upstream regulators of MOMP which includes pro-and anti- apoptosis components that predominantly mediate the mitochondrial (intrinsic) apoptosis pathway 30. Pro-apoptosis proteins Bax and cleaved caspase-3 while anti- apoptosis protein Bcl2 was downregulated as determined by western blot analysis 31.

As mentioned above, a previous finding contradicts with the finding of this study. Thus, we attempted to explore the cause of the discrepancy. We found that under normal conditions, PEA-15 regulates cell proliferation, apoptosis, autophagy, adhesion, migration and glucose metabolism 32. We then focused on apoptosis and proliferation functions influenced by PEA15 in OC as the starting point. This is totally different from the previous study which reported that the antitumor activity of PEA15 was mediated, in part, by the induction of autophagy through the ERK1/2 pathway. In this study, we also explored the effect of PEA15 on ERK and pERK expression and found that silencing of PEA15 decreased the expression of pERK in ovarian cancer cells, this result is consistent with the findings reported by previous studies. We believe that the current understanding of the role played by PEA15 in ovarian carcinoma remains controversial because of its multiple complex functions. It is generally known that 30% of human genes are regulated by miRs which regulate several cellular processes including apoptosis, proliferation and cell cycle 33. Thus, we explored the upstream regulatory miRNA of PEA15. Our bioinformatics analyses and luciferase reporter experiments showed that microRNA212 negatively regulated PEA15 expression. MiR212 has been shown to play important roles in maintaining the viability and function of cancer cells. For instances, a previous study revealed that MiR212 inhibits the proliferation and invasion of human renal cell carcinoma by targeting FOXA1 31. A similar inhibitory effect was found in colon cancer 31. Herein the expression of miR212 was down regulated in ovarian cancer tissues and its overexpression decreased the expression of PEA15 in OC cells.

Furthermore, upregulation of PEA15 abolished the inhibitory effects of miR212 on OC cell lines.

In conclusion, despite the discrepancies observed in our study demonstrated that PEA15 is significantly upregulated in OC tissues, and that PEA15 expression may serve as an independent prognostic marker for patients with OC. In addition, our findings suggest that PEA15 silencing suppresses tumorigenesis in OC cell lines and inhibits cell growth both *in vitro* and *in vivo*. Moreover, PEA15 regulates apoptosis-related proteins and is negatively regulated by miR212. Further studies are warranted to reveal the detailed molecular mechanisms to facilitate the development of new therapeutic targets for OC.

## Supplementary Material

Supplementary figures and tables.

## Figures and Tables

**Table 1:** Correlation of PEA15 expression with patient's clinical and pathological characteristics

Variable	Expression of PEA15		Total	P
	Low	High		
**EOC group**	75	96	171	**0.001**
**Normal**	18	2		
**Total**	93	98	191	
**Histologic subgroups**				
Serous	34	55	89	**0.012**
Endometrioid	10	16	26	
Clear cell	7	7	14	
Mucinous	24	18	42	
Total	75	96	171	
**FIGO stage**				
I	48	57	105	**0.001**
II	15	11	26	
III-IV	12	28	40	
Total	75	96	171	
**Lymph node status**				
-	71	82	153	**0.005**
+	4	14	18	
Total	75	96	171	
**Grade of serous**				
Low	14	20	34	0.46
High	20	35	55	
Total	34	55	89	
**Grade of mucinous**				
1	10	2	12	**0.001**
2	6	8	14	
3	8	8	16	
Total	24	18	42	
**Grade of endometrioid**				
1	3	6	9	0.827
2	3	5	8	
3	4	5	9	
Total	10	16	26	
