# Supplementary material for: The microRNA212 regulated PEA15 promotes ovarian cancer progression by inhibiting of apoptosis
Source: J Cancer. 2020 Jan 13;11(6):1424–35. doi: 10.7150/jca.32886 (PMC6995389; doi:10.7150/jca.32886)
Supplement: Supplementary file 1 — Supplementary figures and tables. [file jcav11p1424s1.pdf]

Supplementary figure1

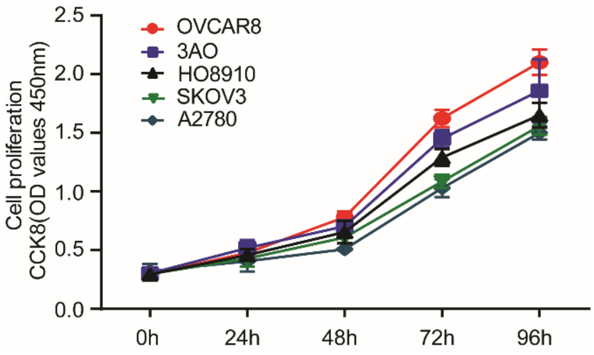

Cell proliferation capacity of OVCAR8, 3AO, SKOV3, A2780, HO8910 cell lines was detected by cck8 assay at 0, 24, 48, 72, 96 h. The result showed that there was no positive correlation between proliferation rate and PEA-15 expression level in the 5 parental cell lines with different expression levels of PEA15.

Supplementary figure2

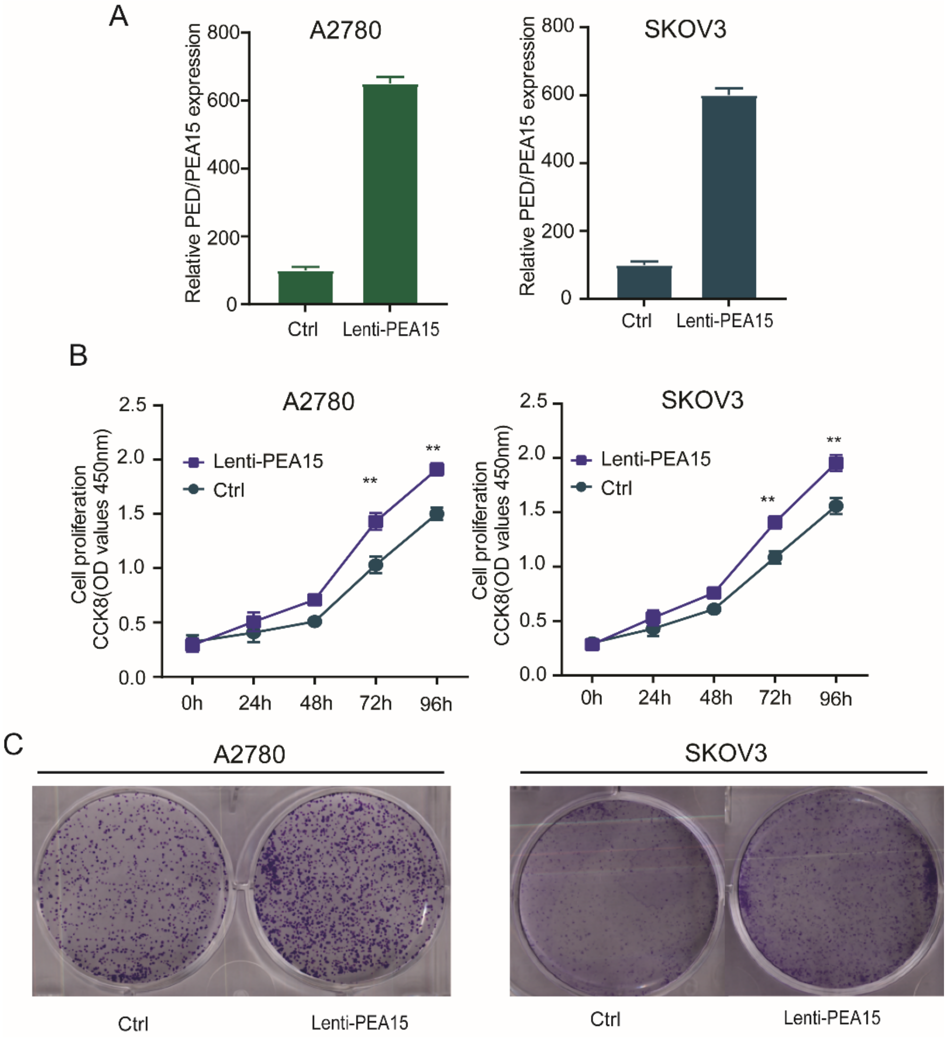

A: PEA15 overexpression efficiency was confirmed by RT-PCR in A2780 and SKOV3 cells.

B: The cell proliferation of Ctrl and Lenti groups in A2780 and SKOV3 cells was evaluated by CCK8 assay at 0, 24, 48, 72, 96 h. The results showed that overexpression of PEA15 significantly promoted the proliferation of A2780 and SKOV3 cells in vitro ($P<0.01$).

C: Plate colony formation assay using A2780/Lenti-PEA15 and SKOV3/ Lenti-PEA15 and Ctrl cells on regular culture plates after 14 days of culture. Relative colony numbers of Lenti-PEA15 cells were significantly higher than that of Ctrl cells.

Supplementary figure3

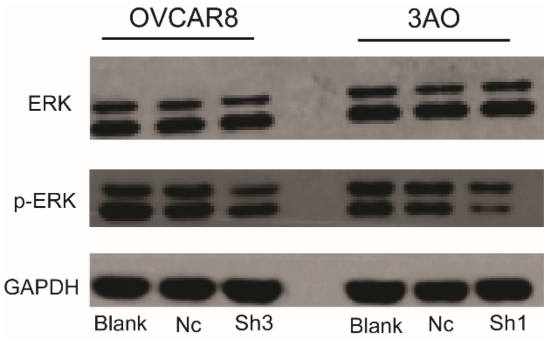

Silencing of PEA15 decreased the expression of pERK in ovarian cancer cells. PEA-15 silencing did not change the expression of ERK, but it decreased the expression of pERK in OVCAR8 and 3AO cells.

.
